# Co-Occurring Alterations of ERBB2 Exon 20 Insertion in Non-Small Cell Lung Cancer (NSCLC) and the Potential Indicator of Response to Afatinib

**DOI:** 10.3389/fonc.2020.00729

**Published:** 2020-05-12

**Authors:** Bo Yuan, Jun Zhao, Chengzhi Zhou, Xiumei Wang, Bo Zhu, Minglei Zhuo, Xilin Dong, Jiemei Feng, Cuihua Yi, Yunpeng Yang, Hua Zhang, Wangyan Zhou, Zhengtang Chen, Sheng Yang, Xinghao Ai, Kehe Chen, Xuefan Cui, Difa Liu, Chunmei Shi, Wei Wu, Yanjun Zhang, Lianpeng Chang, Jin Li, Rongrong Chen, Shuanying Yang

**Affiliations:** ^1^Department of Pulmonary and Critical Care Medicine, The Second Affiliated Hospital of Xi'an Jiaotong University, Xi'an, China; ^2^Key Laboratory of Carcinogenesis and Translational Research (Ministry of Education), Department of Thoracic Medical Oncology-I, Peking University Cancer Hospital and Institute, Beijing, China; ^3^State Key Laboratory of Respiratory Disease, National Clinical Research Center for Respiratory Disease, Guangzhou Institute of Respiratory Health, The First Affiliated Hospital of Guangzhou Medical University, Guangzhou, China; ^4^Department of Oncology, Inner Mongolia Autonomous Region Cancer Hospital, Hohhot, China; ^5^Department of Oncology, Xinqiao Hospital, Chongqing, China; ^6^Department of Respiratory Medicine, Guigang City People's Hospital, Guigang, China; ^7^Department of Medical Oncology, Qilu Hospital of Shandong University, Jinan, China; ^8^Department of Oncology, Sun Yat-sen University Cancer Center, Guangzhou, China; ^9^Department of Oncology, Shaanxi Provincial People's Hospital, Xi'an, China; ^10^Department of Party Affairs, The First Affiliated Hospital of University of South China, Hengyang, China; ^11^Department of Oncology, Fujian Medical University Union Hospital, Fuzhou, China; ^12^Lung Tumor Clinical Medical Center, Shanghai Chest Hospital, Shanghai Jiaotong University, Shanghai, China; ^13^Department of Oncology, The People's Hospital of Guangxi Zhuang Autonomous Region, Nanning, China; ^14^Department of Respiratory Medicine, The First Affiliated Hospital With Nanjing Medical University, Nanjing, China; ^15^Department of Oncology, Haian People's Hospital, Nantong, China; ^16^Department of Thoracic Surgery, The First Hospital Affiliated to AMU (Southwest Hospital), Chongqing, China; ^17^Department of Oncology, Shaanxi Provincial Cancer Hospital, Xi'an, China; ^18^Geneplus-Beijing, Beijing, China

**Keywords:** non-small cell lung cancer, ERBB2 exon 20 insertion, co-occurring alterations, afatinib, clonality status

## Abstract

**Background:** Human epidermal growth factor receptor 2 (ERBB2, HER-2) exon 20 insertion (ERBB2ex20ins) remains a refractory oncogenic driver in lung cancer. So far there is limited data showing the co-occurring mutation background of ERBB2ex20ins in Chinese lung cancer and its relationship with response to afatinib.

**Patients and Methods:** A total of 112 Chinese patients with ERBB2ex20ins identified by next-generation sequencing from 17 hospitals were enrolled. The clinical outcomes of 18 patients receiving afatinib treatment were collected.

**Results:** Among the 112 patients, insertion-site subtypes comprised of A775ins (71%; 79/112), G776indel (17%; 19/112), and P780ins (12%; 14/112). There were 66.1% (74/112) of patients carrying TP53 co-mutation and FOXA1 was the most prevalent co-amplified gene (5.5%, 3/55). The co-occurring genomic feature was similar among three insertional-site subtypes and had an overall strong concordance with the western population from the MSKCC cohort (*R*^2^ = 0.74, *P* < 0.01). For the prognosis, patients with co-occurring mutation in cell-cycle pathway especially TP53 showed shorter OS than patients without [median OS: 14.5 m (95% CI:12.7–16.3 m) vs. 30.3 m (95% CI: not reached), *p* = 0.04], while the OS was comparable among three subtypes. For the response to afatinib, ERBB2ex20ins as a subclonal variant was an independent factor relating to shorter PFS [median PFS: 1.2 m (95% CI: 0.8–1.6 m) vs. 4.3 m (95% CI: 3.3–5.3 m), *p* < 0.05].

**Conclusion:** Our data revealed co-occurring TP53 represent an unfavorable prognosis of patients with ERBB2ex20ins, emphasizing the more valuable role of the co-mutation patterns than insertion-site subtypes in predicting prognosis of this group of patients. Moreover, the clonality status of ERBB2ex20ins was identified as a potential indicator for response to afatinib.

## Introduction

Aberrations in human epidermal growth factor receptor 2 (HER-2, ERBB2) have emerged as oncogenic drivers and therapeutic targets in 1–4% of non-small cell lung cancer (NSCLC) and up to 6% of EGFR/KRAS/ALK-negative lung adenocarcinoma (LUAD) (1, 2). Most of ERBB2 mutations is characterized by inframe insertion occurring at exon 20 in the protein kinase domain (3). Prior studies showed that pan-ERBB family inhibitor (afatinib, dacomitinib) (4, 5), ado-trastuzumab (T-DM1) (6) as well as some new agents such as poziotinib (7), pyrotinib (8) may elicit an objective response in patients with ERBB2 exon 20 insertion (ERBB2ex20ins); however, no therapy has been approved as a standard treatment yet.

Afatinib has been demonstrated its suppressive effect on lung cancer cell lines with ERBB2ex20ins *in vivo* (9). Previous studies also revealed clinical outcomes of afatinib with a 13–19% objective response rate (ORR) and a disease control rate (DCR) around 70% in three separate cohorts (10–12); Nevertheless, there exists profound efficacy heterogeneity on them, such as patients with the same subtype displayed discordant benefits and duration of time.

Several prior studies revealed that genetic co-alterations were independent variables associated with unfavorable prognosis of EGFR-TKIs (13, 14). However, because of its low frequency, researches focused on ERBB2ex20ins have generally been limited to insufficient number of cases from single institution and prevent making a broad assessment of co-existing alteration patterns of ERBB2ex20ins, which may reflect its genomic background heterogeneity and contribute to the variable responsiveness to the targeted therapy. Therefore, making a comprehensive analysis of concomitant mutation spectrum of ERBB2ex20ins in a large cohort and correlating its co-mutation status with prognosis are urgently warranted.

Moreover, growing number of studies are paying attention to the clonality heterogeneity of targetable somatic alterations and adapting the cancer-treatment strategy to taking into account how a tumor evolves (15). It seems that therapy targeted clonal (“trunk”) mutations may be more effective than targeted subclonal (“branch”) ones (16, 17). Nevertheless, how the clonality status of driver aberrations modulates the efficacy of therapy is unclear.

Using the next-generation sequencing (NGS) method, we here described the co-occurring molecular spectrum of ERBB2ex20ins in a cohort of 112 NSCLC patients from 17 hospitals in China. We also compared our spectrum with the western population from Memorial Sloan Kettering Cancer Center (MSKCC) and investigated the impact of co-mutation status on the prognosis of them. Furthermore, we retrospectively assessed the efficacy and tried to identify efficacy predictive factors of afatinib in 18 patients with ERBB2ex20ins.

## Materials and Methods

### Patient Cohort and Clinical Data Collection

**Figure 1:**
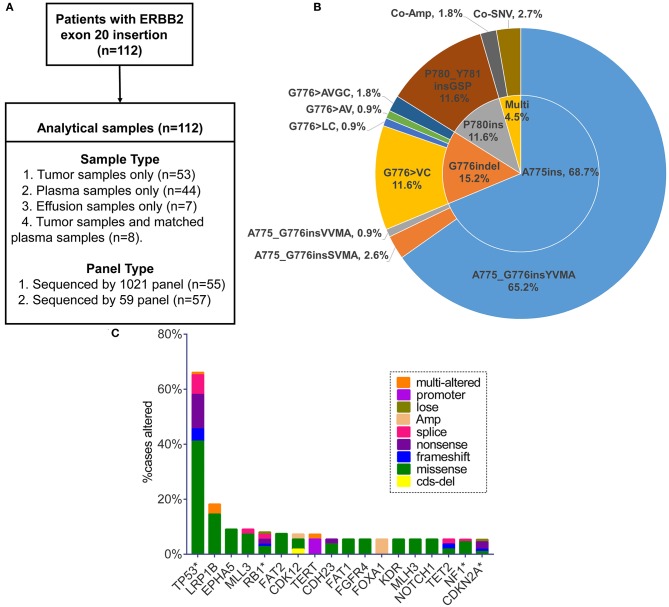
**(A)** Sample types and sequencing panel details of study design. **(B)** Pie chart visualizing eight specified insertion subtypes combined with insertion site. Multi, multiple alterations; Co-Amp, co-amplification; Co-SNV, co-single nucleotide variant. **(C)** Top 18 genes in the highest co-occurring frequency with ERBB2 exon 20 insertion (no relation to the total numbers analyzed). Only the genes with concurrent frequency over 5% are shown. *The genes included in the 59-gene panel.

We retrospectively screened 112 patients (from 17 hospitals) harboring ERBB2ex20ins in a College of American Pathologists (CAP) Laboratory (Geneplus-Beijing, Beijing, People's Republic of China) from July 2016 to December 2018. Samples of tumor tissue, plasma or effusion were analyzed by next-generation sequencing (NGS) assay using two versions (59 or 1,021 cancer-related genes) of capture-based targeted sequencing panel. Gene lists of two versions of sequencing panel are shown in Table S1. The sample type and panel for each patient are shown in Figure 1A. A total of 55 and 57 samples were sequenced using 1,021 or 59-gene panel, respectively. Clinicopathological features were abstracted from the accompanying pathology report submitted by the ordering physician. All patients provided written informed consent for our study. This study was approved by the institutional review board of The Second Affiliated Hospital of Xi'an Jiaotong University and all participating hospital.

The sequencing data of the Memorial Sloan Kettering Cancer Center (MSKCC) Cohort was downloaded from an open-access database named the Cancer Genome Atlas Database, which is publicly available at http://www.cbioportal.org [MSK-IMPACT Clinical Sequencing Cohort (MSKCC, Nat Med 2017)] (18, 19). The data of overall survival (OS) was acquired from the cbioportal website directly. OS was measured from the date when the tumor specimen was collected to the date of death or last follow-up visit (20).

### Response Evaluation

The clinical outcomes of 18 patients treated with afatinib were collected by each contributing doctor in charge and pooled for analysis. Patients were administered afatinib depending on their performance status and other comorbidities at a starting dose of 30, 40, or 50 mg daily. Best response evaluation was assessed according to the Response Evaluation Criteria in Solid Tumors (RECIST, v1.1). The progression free survival (PFS) for afatinib treatment was defined as the time from the start of afatinib treatment to the date of disease progression or death.

### DNA Extraction

Circulating DNA and Genomic DNA for genomic testing were isolated from 3 ml of plasma or effusion and FFPE samples, respectively. Peripheral blood lymphocytes (PBL) DNA were extracted for germline reference (Supplemental Online Methods).

### Target Capture and Next-Generation Sequencing

KAPA Library Preparation Kit (Kapa Biosystems, Wilmington, MA, USA) was applied to prepare Indexed Illumina NGS libraries from peripheral blood lymphocytes (PBL) DNA, and tumor DNA or plasma DNA according to the manufacturer's protocol. Capture probes were designed to cover coding sequences or hot exons of 59 or extended 1,021 genes that are frequently mutated in NSCLC and other common solid tumors (details of sequencing region for each gene are uploaded in Table S1). Libraries were hybridized to custom-designed biotinylated oligonucleotide probes (Integrated DNA Technologies, Iowa, IA, USA). DNA sequencing was performed on the HiSeq 3000 Sequencing System (Illumina, San Diego, CA) with 2 × 101 bp paired-end reads.

### Sequencing Data Analysis

Terminal adaptor sequences and low-quality data were removed from the raw data. The BWA (0.7.12-r1039) was employed to align clean reads to the reference human genome (hg19) (21). MuTect2 (3.4-46-gbc02625) and GATK was applied to call single nucleotide variants (SNVs) and small insertions and deletions (Indels), respectively. Somatic copy number variants (CNVs) were identified using CONTRA (2.0.8) (22). Moreover, we employed the NoahCare Tool Kit using NCfilter (software developed by self, version 1.5.0) for fastq data QC, NCbamInfo (version 0.2.0) for alignment QC, NCanno (version 0.1.1) for annotation with multiple databases, and NChot (version 0.1.0) for hotspot region variant review and recall. All final candidate variants were all manually verified using the Integrative Genomics Viewer (IGV) Browser.

### Clonality Analysis

The subclonal architecture of all DNA samples were constructed by PyClone run with 20,000 interactions and default parameters (23). Variants were clustered as previously described (23), briefly, the copy number information of each SNV was used as input for PyClone analysis (24, 25), and the cancer cell fraction (CCF) was inferred. Variants located in the cluster with greatest mean CCF were defined as clonal, the rest were subclonal (23).

### Statistical Analysis

All statistical analyses were performed using SPSS version 19.0 (SPSS Company, Chicago, IL). Categorical and continuous variables were compared by Fisher's exact test and Kruskal-Wallis *H*-test, respectively. The Pearson correlation coefficient was applied to assess the linear correlation degree of co-occurring genes' frequency appearing in Our Cohort and MSKCC Cohort. The OS and PFS were estimated using the Kaplan-Meier method and compared with the log-rank test. A multi-variant regression model was calculated for HRs and 95% CIs. All statistical tests were two-sided, and *p* < 0.05 was defined as statistical significance.

## Result

### Patients With ERBB2 exon 20 Insertion

One hundred and twelve patients carrying ERBB2ex20ins were screened from July 2016 to December 2018 (Figure 1A). The clinical characteristics for these patients were summarized in Table 1. In all, patients were predominantly in the stage IV (80/112, 72%) and had the histology of adenocarcinoma (68%,76/112). There were slightly more female (54%; 60/112) than male (46%; 52/112), with a median age of 61.5 years (range: 28–87 years).

**Table 1:** Clinical characteristics of patients with ERBB2 exon 20 insertion in different positions.

**Characteristics**	**A775ins (*n* = 79)**	**G776indel (*n* = 19)**	**P780ins (*n* = 14)**	**Sum**	***P*-value**
**Age at initial diagnosis**					
Median (range)	62 (28–83)	58 (29–87)	64 (48–83)	61.5 (28–87)	0.425
Unknown	6	0	2	8	
**Gender**					
Female	39 (49%)	11(58%)	10 (71%)	60 (54%)	0.287
Male	40 (51%)	8 (42%)	4 (29%)	52 (46%)	
**Histology**					
NSCLC NOS	14 (18%)	3 (16%)	1 (7%)	18 (16%)	nc
Adenocarcinoma	55 (70%)	11(58%)	10 (72%)	76 (68%)	
Squamous carcinoma	0	0	1(7%)	1(1%)	
Unknown	10 (12%)	5 (26%)	2 (14%)	17 (15%)	
**Stage**					
I–III	15 (19%)	2 (10%)	2 (14%)	19 (16%)	0.850
IV	55 (70%)	14 (74%)	11 (79%)	80 (72%)	
Unknown	9 (11%)	3 (16%)	1 (7%)	13 (12%)	

*P-values are calculated with Fisher's exact test except for age using the Kruskal-Wallis H-test*.

*ins, insertion; indel, insertion and deletion; NSCLC NOS, non-small cell lung cancer not other specified; nc, not calculate*.

Totally, eight specific insertion types of ERBB2ex20ins were identified. Considering the fact that certain studies discussing the efficacy of targeted therapy for ERBB2ex20ins are always based on the different insertion sites, we classified them into three subtypes. The most common subtype was four amino acids insertion at codon 775 (A775ins; 70.5%), followed by insertion combined with deletion occurring at codon 776 (G776indel; 17.0%), and three amino acids insertion at codon 780 (P780ins; 12.5%). Multi-alterations were present in five patients, with two patients harboring concurrent ERBB2 amplification and three patients carrying ERBB2ex20ins with ERBB2 single nucleotide variant (SNV) referring to p.A775_G776insYVMA+ p.R897Q, p.P780_Y781insGSP+ p.G519R, and p.G776delinsAVGC+ p.G776A (Figure 1B).

Moreover, insertion-subtype abundance was not significantly different among the sample types (*p* = 0.41; Table S2). It indicated that distinct sample type was not biased toward the detection of certain ERBB2 subtype.

### Co-occurring Genomic Profile of ERBB2 exon 20 Insertion

#### Spectrum of Co-occurring Alterations and Characteristics in Different Insertion Sites

On the basis of 59 genes strongly associated with cancer, 80.4% (90/112) of patients had at least one additional alteration, with 48.9% (44/90) of them carrying one and 27.8% (25/90) carrying two. Three or more concomitant alterations were present at the rest of 23.3% (21/90) patients. TP53 was the most frequent gene co-mutant with ERBB2ex20ins, making up 66.1% (74/112) cases, with predominant alteration type of missense mutation (63.5%, 47/74), concentrating on exon 5, 8, 6, 7 (76.7%, 56/74; range: exon 4-exon 11) (Figure S1A, Table S3). The remaining prevalent co-occurring genes were LRP1B (18.2%, 10/55), EPHA5 (9.1%, 5/55), MLL3 (9.1%, 5/55), and RB1 (8.0%; 9/112) (no relation to the total numbers analyzed). FOXA1 appeared in 5.5% (3/55) of patients and became the most common co-mutant gene in the form of amplification (Figure 1C). Putative driver aberrations including EGFR (L858R or 19del), ROS1 fusions, ALK receptor tyrosine kinase gene (ALK) rearrangement, KRAS, BRAF (V600E) were not found in this cohort, probably mutually exclusive from ERBB2ex20ins.

**Figure 2:**
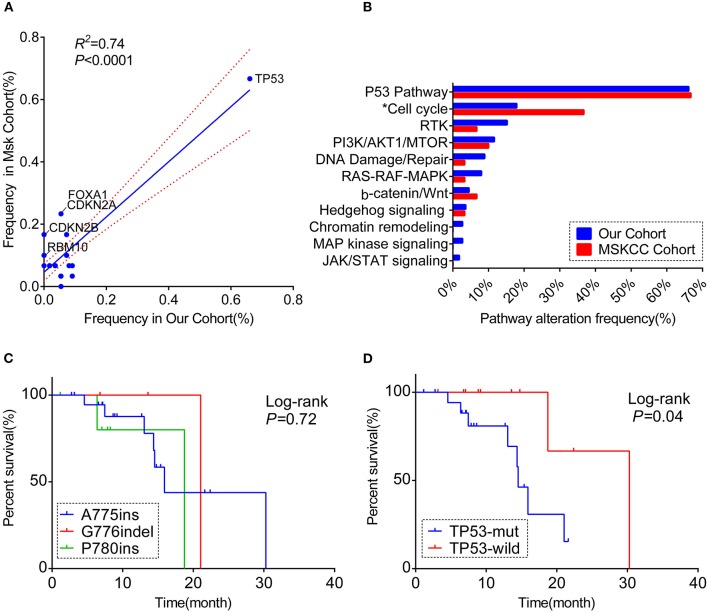
A comprehensive comparison of the co-occurring profile between our cohort and MSKCC cohort for the frequency of **(A)** totally matched 28 genes (the genes included in the analysis were matched in both cohorts' panel with the frequency over 5%; the genes labeled are significantly different between the two cohorts). **(B)** Pathway enriched (Only genes included in 59-gene panel were analyzed; *the pathway significantly different between the two cohorts). RTK, receptor tyrosine kinase/growth factor signaling. **(C)** Kaplan–Meier curve showing the difference of median overall survival (OS) among patients harboring three different ERBB2 insertion subtypes. **(D)** Kaplan–Meier curve visualizing the effect of TP53 alteration on OS.

Of the pathway level, we classified the co-mutant genes according to the pathway involved. 86.7% (78/90) of patients, who carried at least one additional mutation, had the co-altered genes enriched in the cell cycle, followed by receptor tyrosine kinase/growth factor signaling (RTK) (15.2%) and DNA damage/repair (8.9%) (Figure 2B). Furthermore, some patients had co-mutant genes involved multiple important pathways simultaneously, while some patients had more than one co-mutant genes involving one single pathway (Table S4).

We also explored the co-occurring alteration feature among A775ins (*n* = 79), G776indel (*n* = 19), and P780ins (*n* = 14). No substantial discrepancy was observed among the three groups at either the co-occurring somatic alterations (only TP53 was included in the analysis) or pathway enriched, the clinical details were as well (Figures S2A,C, Table 1). The location and exon distribution of TP53 mutation were comparable in three insertion-site subtypes (Figures S1B–E); however, when considering TP53 mutation types, there was a tendency that G776indel may be less adept at co-occurring with TP53 missense mutations, with no statistically significant (*p* = 0.06; Figure S2B).

#### Spectrum Comparison With the Western Population From the MSKCC Cohort

Next, we compared our data with the findings previously reported by the MSKCC, which included 1,563 tumor specimens from patients with NSCLC. Totally 30 patients harboring ERBB2ex20ins involved in this cohort.

Overall, both the proportion of three subtypes and the whole molecular co-occurring mutation spectrum (genes that co-altered at a frequency over 5% in each cohort) were similar between the two cohorts (*R*^2^ = 0.74, *P* < 0.01), although the co-mutant frequencies of certain genes were higher in the MSKCC cohort than in ours(Figure 2A, Table S5). Notably, these genes in slightly higher frequency were in the form of copy number variant (CNV) (Figure S2D), and it is probably caused by the low detection rate of CNV due to the mixed plasma samples in our samples.

For the pathway analysis, the enrichment of each pathway in our cohort was in accord with the MSKCC cohort, with a slightly higher frequency of cell-cycle pathway enriched in MSKCC cohort (36.7 vs. 17.9%, *P* = 0.044) on account of the higher frequency of CDKN2A alteration (Figure 2B, Table S4).

### Impact of Insertional-Site Subtypes and Co-occurring Mutational Status on OS

Based on the complete overall survival (OS) from the MSKCC cohort, prognosis impact of ERBB2 insertion-site subtypes and genes co-occurring over 5 or more cases in either cohort were evaluated. Statistic descriptive of co-occurring genes included in the analysis were summarized in Figure S3A. There was a trend that patients harboring co-occurring genes enriched in cell-cycle pathway showed a worse survival, with no significantly statistic difference (*p* = 0.059; Figure S3B). However, worse overall survival was seen in patients with co-mutations in TP53 [median OS:14.5 m (95% CI:12.7–16.3 m) vs. 30.3 m (95% CI: not reached); log-rank test], while OS was not significantly different among three subtypes (*p* = 0.72, Figures 2C,D).

Prior study revealed TP53 mutation in exons 5, 7, 8, and 9 sharing a better prognosis than other sites in the advanced NSCLC (26). In this regard, we investigate the prognosis value of co-occurring TP53 mutation in exons 5, 7, 8, and 9 and whether they can be even more relevant in a specific subgroup of patients with ERBB2ex20ins mutation (i.e., A775ins, G776indel, and P780ins subgroups) but found negative result (log rank, *p* = 0.095; Fisher exact test, *p* = 0.427; Figures S3C,D).

### Clinical Outcomes of Afatinib for Patients Harboring ERBB2ex20ins

#### Afatinib Treatment Efficacy Overview

The basic clinical and molecular characteristics of 18 patients treated with afatinib were summarized in Table 2. Nearly all of patients were in the advanced stage and 61.1% (12/18) of patients receiving afatinib as 2 line or more.

**Table 2:** Clinical and molecular characteristics of patients treated with afatinib.

**Characteristics**	**NCB (*n* = 8)**	**DCB (*n* = 10)**	**Sum (*n* = 18)**	***P*-value**
**Age at initial diagnosis, years**				
Median (range)	56.5 (40–75)	54 (29–69)	55.5 (29.75)	
<65	5	9	14 (77.8%)	0.28
≥65	3	1	4 (22.2%)	
**Gender**				
Female	5	5	10 (55.6%)	0.66
Male	3	5	8 (44.4%)	
**Tobacco use**				
Never	5	4	9 (50.0%)	0.15
Former or current	3	2	5 (27.8%)	
Unknown	0	4	4 (22.2%)	
**Histology**				
Adenocarcinoma	8	10	18 (100.0%)	
**Brain metastasis**				
Yes	3	4	7 (38.9%)	0.34
No	3	6	9 (50.0%)	
NA	2	0	2 (11.1%)	
**Tumor stage**				
IIIa	1	0	1 (5.6%)	nc
IV	6	10	16 (88.8%)	
Unknown	1	0	1 (5.6%)	
**No. line of afatinib treatment**				
1	3	3	6 (27.8%)	1.00
≥2	5	7	12 (61.1%)	
**ERBB2ex20ins subtypes**				
A775 insertion	6	7	13 (72.2%)	1.00
G776 indel	1	2	3 (16.7%)	
P780 insertion	1	1	2 (11.1%)	
**Concurrent TP53 alteration**				
Yes	7	6	13 (72.2%)	0.31
No	1	4	5 (27.8%)	
**Concurrent TP53 missense mutation**				
Yes	6	4	10 (55.6%)	0.19
No	2	6	8 (44.4%)	

*DCB, durable clinical benefit; NCB, no durable benefit; nc, not calculate*.

**Figure 3:**
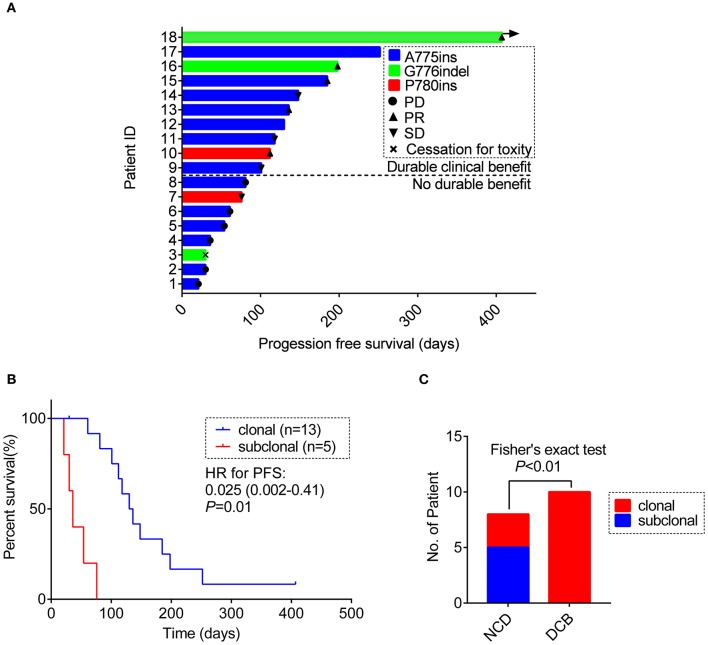
**(A)** Swimming plot visualizing the response details for afatinib in each patient (*n* = 18). **(B)** Kaplan-Meier comparing PFS for ERBB2ex20ins as clonal or subclonal variant (*p*-values determined by multi-variant regression analysis and HR with 95% CI are shown). **(C)** The distribution of ERBB2 clonality status between the DCB group and the NDB group. DCB, durable clinical benefit; NDB, no durable benefit.

Of the 18 patients, tumor remission data according to RECIST 1.1 criteria were available for 15 patients. Among them, 5 patients achieved PR (33.3%) and 4 patients achieved SD (26.7%). All PRs were, respectively, observed in 3 separate insertion subtypes, whereas the patients with PD only involved in the subtype of A775ins. The median time on treatment with afatinib was 3.7 months (95% CI: 2.1–5.3 m; range: 0.7–13.4 m). The median duration time for patients responding to afatinib was 4.5 months (95% CI: 3.6–5.4 m; range: 2.5 m−13.4 months). The response details and duration of response (DoR) of afatinib for each patient were showed in Figure 3A. One patient harboring G776delinsVC was treated for afatinib as first-line therapy with PR for over 13.4 months and didn't achieve disease progression until the last follow-up in this study. As for the rest of patients, 3 cases (16.7%) responding to afatinib had a DoR over 6 months.

#### Impact of Clonality Status and Co-occurring Mutations of ERBB2ex20ins on Afatinib Treatment Outcome

For 18 patients treated with afatinib, we identified 54 somatic SNVs, 4 CNVs and 18 somatic indels in 18 samples, for an average of 4.3 somatic variants per sample. In this regard, we applied method PyClone to evaluate whether the ERBB2ex20ins carried by the patients were clonal or subclonal mutations.

Result revealed that ERBB2ex20ins as subclonal variants was significantly associated with shorter PFS of afatinib [median PFS: 1.2 m (95% CI: 0.8–1.6 m) vs. 4.3 m (95% CI: 3.3–5.3 m), *p* < 0.01], while co-occurring TP53 mutation and insertion-site subtypes had no significant impact on the efficacy of afatinib. This result remained significant when adjusted for ERBB2 insertion subtypes, TP53 mutation, TP53 missense mutation and no. line of afatinib [HR: 0.025 (0.002–0.41); *p* = 0.01)] (Figure 3B, Table S6).

Next, we divided the patients into durable clinical benefit (DCB) cohort and no durable benefit (NDB) cohort (The DCB was defined as the patients achieving PR or SD and having the duration of PFS for over 3 months; the NDB referred to the patients having the PFS <3 months). Clinical baseline parameters for two groups of patients are displayed in Table 2 and basically similar among each parameter. The variables including concurrent TP53 mutation and TP53 missense mutation were not significantly different between the two groups (data not show, Fisher's exact test, *p* > 0.05) except for the ERBB2ex20ins clonality status (Fisher exact test, *p* < 0.01; Figure 3C).

#### Dynamic Detection and Afatinib Resistance

To gain some insight into the potential mechanism upon afatinib resistance, we analyzed two patients conducting NGS at two time points in the course of afatinib treatment. The clinical characteristics and test details for two patients were summarized in Table S7. Both patients were non-smoking female. For the Patient#1, ERBB2 amplification [copy number (CN) = 3.1] occurred in the repeat biopsy sample upon the progression of afatinib. Similarly, the patient#2 also presented the ERBB2 amplification (CN = 2.74) which was undetected in the initial plasma sample after taking afatinib for half of a month, unfortunately, we did not examine the ERBB2 CN status at the time of progression on afatinib. Despite this, it can be speculated that ERBB2 amplification may represent a potential resistance mechanism of afatinib.

## Discussion

In this study, we delineate the co-occurring alterations and common pathway involved addicted to ERBB2ex20ins in a representative NSCLC cohort of 112 patients and correlate co-mutation patterns with the prognosis of patients harboring ERBB2ex20ins. Moreover, to our knowledge, we present the first time to examine the impact of clonality status of oncogenic drivers in relation to the efficacy of targeted therapy.

The recent widespread use of NGS enables us to move researches from concentrating solely on the driver gene to the full view of genomic co-alterations, which may have prognostic implications. To date, somatic mutations in TP53 are the most prevalent co-mutation in EGFR-mutant lung adenocarcinoma (LUAD) with a frequency of 54.6–64.6% and several studies have identified TP53 co-alteration as a negative prognosis marker, with consistently predicting worse clinical outcomes receiving EGFR-TKI therapy (27). In our study, TP53 ranked as the most common accompanying somatic altered gene with a frequency of 66%; this frequency was slightly higher than the previously reported 51.6% (10), possibly due to the fewer proportion of female in our cohort (28). Our results showed that patients had a worse OS when co-occurring mutation in TP53, which is also validated in a previous study (29). Recently, the different prognosis value was recognized in distinct exons and alteration types of TP53 mutation and the results were inconsistent. Exons 5, 7, 8 and 9 were reported to share a better prognosis than other sites (26); it is worth mentioning that the study referred here sought to reveal the prognostic value of TP53 alterations in advanced NSCLC compared to most of studies limited to the early stage or EGFR-mutant background (30, 31). Unfortunately, TP53 mutations in exons 5, 7, 8, and 9 did not produce more favorable prognosis than other sites in the advanced NSCLC patients carrying ERBB2 mutation. Clinicopathological characteristics and treatment status should be well-defined to clearly investigate the prognosis impact of various TP53 exons. Moreover, there is a tendency that G776indel subtype may be less adept at co-occurring with TP53 missense mutations, however, whether this characteristic will have a beneficial effect on the prognosis for them remains to be explored. Interestingly, we found neither the co-mutant frequency of TP53 nor pathway enrichment was significantly different among three insertion subtypes, and the OS was comparable as well. For the clinical practice, we suppose that the co-occurring mutation status may have greater impact on the prognosis for this subset of patients than the insertion subtypes itself. Moving forward, the study highlighted multiple concurrent mutations besides ERBB2 insertion subtypes should be tested prospectively in order to provide better predictions of prognosis for them.

In order to systematically understand the co-mutation profile of ERBB2ex20ins, we cataloged co-altered genes based on existing biological pathway knowledge and the cell-cycle (86.7%), receptor tyrosine kinase/growth factor signaling (RTK) (15.2%) and DNA Damage/Repair (8.9%) showed predominance among all the involved pathways. Prior study found that cell-cycle and DNA-damage response pathway are involved in leptomeningeal metastasis of NSCLC (32). This finding was somehow inter-correspondent with the likely unfavorable prognosis for the patients with the co-occurring genes enriched in the cell-cycle pathway in our study, although the survival discrepancy was not significant maybe on account of the insufficient follow-up time or limited sub-group sample size. Unfortunately, we cannot collect the detailed metastasis status for each patient; however, for the patients treated with afatinib, we found seven in 16 of patients having brain metastasis. Moreover, a recent retrospective study found patients carrying ERBB2 mutations in lung cancer developed more brain metastases on treatment than patients with KRAS mutations [28 vs. 8%; hazard ratio (HR), 5.2; *p* < 0.001] and trended more than patients with EGFR mutations [28 vs. 16%; HR, 1.7; *p* = 0.06; (33)]. These findings may underline the central nervous system (CNS) surveillance practices in patients with ERBB2 alterations and the urgent need for the development of novel HER2-targeted agents with active efficacy in the CNS.

The co-occurring genomic spectrum of ERBB2ex20ins in our cohort of Chinese people had an overall strong concordance with the MSKCC cohort from the United States (*R*^2^ = 0.74, *p* < 0.01). In a retrospective study collated two cohorts of patients with ERBB2 alteration from the MSKCC and Guangdong General Hospital, they also found great consistency with each other in the aspect of the prevalence and baseline clinical parameters of patients possessing ERBB2 mutation (34). These findings, on the one hand, can be supporting evidence for U.S.-China collaborations in clinical trials to accelerate new drug development for this infrequent mutation; on the one hand, highlight the robustness of our results.

An important aspect of our study is that we found the clonality status of ERBB2ex20ins was an independent potential indicator for response to afatinib. It is well-known that there exists substantial intratumor heterogeneity and tumor evolves in a trunk-branch model. The “trunk” mutation (clonal mutation) was known as taking place in the early development of cancer and expected to present in every tumor subclone and region, whereas the mutation defined as “branch” would present in a certain fraction of tumor cells and regions (17). Thus, alterations closer to the clonal variant were associated with numerical greater variant allele frequency (VAF). Driver mutations in lung cancer can occur both clonally and subclonally (35). Rachiglio et al. (36) reported that patients harboring EGFR alteration in a lower VAF presented shorter PFS than not, to some extent, this reflecting the clonality status may affect the efficacy of TKIs. These lead us to speculate that other small molecule TKIs in other molecularly defined cohorts may be even more efficacious when targeting the driver mutation as the clonal variants. In this study, however, we should make this result conclusive with caution due to the limited sample size, further exploration in a large cohort named DARWIN trial (Deciphering Anti-tumor Response With intratumor Heterogeneity; Clinical Trials No. NCT02183883) is ongoing (37, 38).

ERBB2 amplification has been identified as a resistance mechanism induced upon treatment with erlotinib or gefitinib (39, 40) and was observed in 12% of tumor samples obtained from patients at resistance to EGFR TKI therapy (41); however, its role in afatinib resistance is unclear. Chuang et al. (42) reported a patient carrying ERBB2ex20ins, whose plasma samples were obtained upon progression on her initial chemotherapy, erlotinib and afatinib, and results showed that the ERBB2 copy number (CN) level increased over time. In our study, we also found two patients treated with afatinib acquired ERBB2 CN gain after taking afatinib for half of a month and upon gained resistance; notably, for the first patient, the biopsy from the initial lesion was taken, respectively, before and upon progression on afatinib, which makes the result more reliable. This makes us speculate that the patients carrying combined ERBB2 mutation and amplification may be less benefit from afatinib. Further basic research may explore this hypothesis.

Admittedly, our study exists several limitations. Firstly, the sequencing panel in the cohort is not uniform, which impeded us making the deep understanding of co-occurring landscape of ERBB2ex20ins, and we can only analyze co-mutant feature of TP53 among the three insertion-site subgroups. Large-panel NGS should be conducted uniformly in further studies when enrolling patients for the research. Another limitation is due to its retrospective nature, a small sample size of the study, selected bias and various imaging intervals are inevitable in the process of assessing the clinical outcomes of afatinib; nonetheless, the ORR and PFS of afatinib treatment was almost correlated well with prior studies. Furthermore, since we used single tumor sample taken at a one-time point in the disease course, we may not verify the true clonality status of each mutation; however, this single-point samples may be more likely to underestimate the true extent of heterogeneity within tumors rather than distinguishing clonal from subclonal variants. Importantly, although multifocal or repeated tumor biopsies is better for tracking the true evolution process of tumor development, single sampling may be easier to achieve in the clinical practice.

In summary, our data revealed co-occurring TP53 represent an unfavorable prognosis of patients with ERBB2ex20ins, highlighting the greater impact of the co-mutation patterns than insertion-site subtypes on the prognosis of this group of patients. Furthermore, our clinical outcome data for afatinib confirmed its certain efficacy for patients with ERBB2ex20ins and suggested the clonality status of ERBB2 mutation may be a potential indicator of response to afatinib.

## Data Availability Statement

The original contributions presented in the study are publicly available. This data can be found here: European Variation Archive (EVA) (www.ebi.ac.uk/eva) (PRJEB37583).

## Ethics Statement

The study involving human participants were reviewed and approved by the institutional review board of The Second Affiliated Hospital of Xi'an Jiaotong University and all participating hospitals (including Peking University Cancer Hospital and Institute, The First Affiliated Hospital of Guangzhou Medical University, Inner Mongolia Autonomous Region Cancer Hospital, Xinqiao Hospital, Guigang City People's Hospital, Qilu Hospital of Shandong University, Sun Yat-sen University Cancer Center, Shaanxi Provincial People's Hospital, The First Affiliated Hospital of University of South China, Fujian Medical University Union Hospital, Shanghai Chest Hospital, The People's Hospital of Guangxi Zhuang Autonomous Region, The First Affiliated Hospital with Nanjing Medical University, Haian People's Hospital, The First Hospital Affiliated to AMU (Southwest Hospital), Shaanxi Provincial Cancer Hospital).

## Author Contributions

ShuY, LC, BY, and JZ designed the study. BY, JZ, JL, and RC contributed to data analysis. BY and JZ prepared the manuscript of the study. CZ, XW, BZ, MZ, XD, JF, CY, YY, HZ, WZ, ZC, SheY, XA, KC, XC, DL, CS, WW, and YZ collected the samples and assembled the clinical data. All authors read and approved the final manuscript.

## Conflict of Interest

The authors declare that the research was conducted in the absence of any commercial or financial relationships that could be construed as a potential conflict of interest.

## References

[1] Arcila ME Chaft JE Nafa K Roy-Chowdhuri S Lau C Zaidinski M. Prevalence, clinicopathologic associations, and molecular spectrum of ERBB2 (HER2) tyrosine kinase mutations in lung adenocarcinomas. Clin Cancer Res. (2012) 18:4910–8. 10.1158/1078-0432.CCR-12-0912 22761469 PMC3865806

[2] Mazieres J Peters S Lepage B Cortot AB Barlesi F Beau-Faller M. Lung cancer that harbors an HER2 mutation: epidemiologic characteristics and therapeutic perspectives. J Clin Oncol. (2013) 31:1997–2003. 10.1200/JCO.2012.45.6095 23610105

[3] Shigematsu H Takahashi T Nomura M Majmudar K Suzuki M Lee H. Somatic mutations of the HER2 kinase domain in lung adenocarcinomas. Cancer Res. (2005) 65:1642–6. 10.1158/0008-5472.CAN-04-4235 15753357

[4] Mazieres J Barlesi F Filleron T Besse B Monnet I Beau-Faller M. Lung cancer patients with HER2 mutations treated with chemotherapy and HER2-targeted drugs: results from the European EUHER2 cohort. Ann Oncol. (2016) 27:281–6. 10.1093/annonc/mdv573 26598547

[5] Kris MG Camidge DR Giaccone G Hida T Li BT O'Connell J. Targeting HER2 aberrations as actionable drivers in lung cancers: phase II trial of the pan-HER tyrosine kinase inhibitor dacomitinib in patients with HER2-mutant or amplified tumors. Ann Oncol. (2015) 26:1421–7. 10.1093/annonc/mdv186 25899785 PMC5006511

[6] Li BT Shen R Buonocore D Olah ZT Ni A Ginsberg MS. Ado-trastuzumab emtansine for patients with HER2-mutant lung cancers: results from a phase II basket trial. J Clin Oncol. (2018) 36:2532–7. 10.1200/JCO.2018.77.9777 29989854 PMC6366814

[7] Robichaux JP Elamin YY Tan Z Carter BW Zhang S Liu S. Mechanisms and clinical activity of an EGFR and HER2 exon 20-selective kinase inhibitor in non-small cell lung cancer. Nat Med. (2018) 24:638–46. 10.1038/s41591-018-0007-9 29686424 PMC5964608

[8] Wang Y Jiang T Qin Z Jiang J Wang Q Yang S. HER2 exon 20 insertions in non-small-cell lung cancer are sensitive to the irreversible pan-HER receptor tyrosine kinase inhibitor pyrotinib. Ann Oncol. (2019) 30:447–55. 10.1093/annonc/mdy542 30596880 PMC7360147

[9] Suzawa K Toyooka S Sakaguchi M Morita M Yamamoto H Tomida S. Antitumor effect of afatinib, as a human epidermal growth factor receptor 2-targeted therapy, in lung cancers harboring HER2 oncogene alterations. Cancer Sci. (2016) 107:45–52. 10.1111/cas.12845 26545934 PMC4724821

[10] Liu Z Wu L Cao J Yang Z Zhou C Cao L. Clinical characterization of ERBB2 exon 20 insertions and heterogeneity of outcomes responding to afatinib in Chinese lung cancer patients. Onco Targets Ther. (2018) 11:7323–31. 10.2147/OTT.S173391 30425522 PMC6205822

[11] Peters S Curioni-Fontecedro A Nechushtan H Shih JY Liao WY Gautschi O. Activity of afatinib in heavily pretreated patients with ERBB2 mutation-positive advanced NSCLC: findings from a global named patient use program. J Thorac Oncol. (2018) 13:1897–905. 10.1016/j.jtho.2018.07.093 30096481

[12] Lai WV Lebas L Barnes TA Milia J Ni A Gautschi O. Afatinib in patients with metastatic or recurrent HER2-mutant lung cancers: a retrospective international multicentre study. Eur J Cancer. (2019) 109:28–35. 10.1016/j.ejca.2018.11.030 30685684 PMC6426688

[13] Blakely CM Watkins TBK Wu W Gini B Chabon JJ McCoach CE. Evolution and clinical impact of co-occurring genetic alterations in advanced-stage EGFR-mutant lung cancers. Nat Genet. (2017) 49:1693–704. 10.1038/ng.3990 29106415 PMC5709185

[14] Hong S Gao F Fu S Wang Y Fang W Huang Y. Concomitant genetic alterations with response to treatment and epidermal growth factor receptor tyrosine kinase inhibitors in patients with egfr-mutant advanced non-small cell lung cancer. JAMA Oncol. (2018) 4:739–42. 10.1001/jamaoncol.2018.0049 29596544 PMC5885210

[15] Willyard C. Cancer therapy: an evolved approach. Nature. (2016) 532:166–8. 10.1038/532166a 27075079

[16] Lohr JG Stojanov P Carter SL Cruz-Gordillo P Lawrence MS Auclair D. Widespread genetic heterogeneity in multiple myeloma: implications for targeted therapy. Cancer Cell. (2014) 25:91–101. 10.1016/j.ccr.2013.12.015 24434212 PMC4241387

[17] Yap TA Gerlinger M Futreal PA Pusztai L Swanton C. Intratumor heterogeneity: seeing the wood for the trees. Sci Transl Med. (2012) 4:127ps10. 10.1126/scitranslmed.3003854 22461637

[18] Cerami E Gao J Dogrusoz U Gross BE Sumer SO Aksoy BA. The cBio cancer genomics portal: an open platform for exploring multidimensional cancer genomics data. Cancer Dis. (2012) 2:401–4. 10.1158/2159-8290.CD-12-0095 22588877 PMC3956037

[19] Gao J Aksoy BA Dogrusoz U Dresdner G Gross B Sumer SO. Integrative analysis of complex cancer genomics and clinical profiles using the cBioPortal. Sci Signal. (2013) 6:pl1. 10.1126/scisignal.2004088 23550210 PMC4160307

[20] Zehir A Benayed R Shah RH Syed A Middha S Kim HR. Mutational landscape of metastatic cancer revealed from prospective clinical sequencing of 10,000 patients. Nat Med. (2017) 23:703–13. 10.1038/nm.4333 28481359 PMC5461196

[21] Li H Durbin R. Fast and accurate short read alignment with burrows-Wheeler transform. Bioinformatics. (2009) 25:1754–60. 10.1093/bioinformatics/btp324 19451168 PMC2705234

[22] Li J Lupat R Amarasinghe KC Thompson ER Doyle MA Ryland GL. CONTRA: copy number analysis for targeted resequencing. Bioinformatics. (2012) 28:1307–13. 10.1093/bioinformatics/bts146 22474122 PMC3348560

[23] Roth A Khattra J Yap D Wan A Laks E Biele J. PyClone: statistical inference of clonal population structure in cancer. Nat Methods. (2014) 11:396–8. 10.1038/nmeth.2883 24633410 PMC4864026

[24] Jamal-Hanjani M Wilson GA McGranahan N Birkbak NJ Watkins TBK Veeriah S. Tracking the evolution of non-small-cell lung cancer. N Engl J Med. (2017) 376:2109–21. 10.1056/NEJMoa1616288 28445112

[25] Murtaza M Dawson SJ Pogrebniak K Rueda OM Provenzano E Grant J. Multifocal clonal evolution characterized using circulating tumour DNA in a case of metastatic breast cancer. Nat Commun. (2015) 6:8760. 10.1038/ncomms9760 26530965 PMC4659935

[26] Jiao XD Qin BD You P Cai J Zang YS. The prognostic value of TP53 and its correlation with EGFR mutation in advanced non-small cell lung cancer, an analysis based on cBioPortal data base. Lung Cancer. (2018) 123:70–5. 10.1016/j.lungcan.2018.07.003 30089598

[27] Skoulidis F Heymach JV. Co-occurring genomic alterations in non-small-cell lung cancer biology and therapy. Nat Rev Cancer. (2019) 19:495–509. 10.1038/s41568-019-0179-8 31406302 PMC7043073

[28] Ma X Le Teuff G Lacas B Tsao MS Graziano S Pignon JP. Prognostic and predictive effect of TP53 mutations in patients with non-small cell lung cancer from adjuvant cisplatin-based therapy randomized trials: a LACE-bio pooled analysis. J Thorac Oncol. (2016) 11:850–61. 10.1016/j.jtho.2016.02.002 26899019

[29] Tomizawa K Suda K Onozato R Kosaka T Endoh H Sekido Y. Prognostic and predictive implications of HER2/ERBB2/neu gene mutations in lung cancers. Lung Cancer. (2011) 74:139–44. 10.1016/j.lungcan.2011.01.014 21353324

[30] Canale M Petracci E Delmonte A Chiadini E Dazzi C Papi M. Impact of TP53 mutations on outcome in EGFR-mutated patients treated with first-line tyrosine kinase inhibitors. Clin Cancer Res. (2017) 23:2195–202. 10.1158/1078-0432.CCR-16-0966 27780855

[31] Labbe C Cabanero M Korpanty GJ Tomasini P Doherty MK Mascaux C. Prognostic and predictive effects of TP53 co-mutation in patients with EGFR-mutated non-small cell lung cancer (NSCLC). Lung Cancer. (2017) 111:23–9. 10.1016/j.lungcan.2017.06.014 28838393

[32] Fan Y Zhu X Xu Y Lu X Xu Y Wang M. Cell-cycle and DNA-damage response pathway is involved in leptomeningeal metastasis of non-small cell lung cancer. Clin Cancer Res. (2018) 24:209–16. 10.1158/1078-0432.CCR-17-1582 29030356

[33] Offin M Feldman D Ni A Myers ML Lai WV Pentsova E. Frequency and outcomes of brain metastases in patients with HER2-mutant lung cancers. Cancer. (2019) 125:4380–87. 10.1002/cncr.32461 31469421 PMC6891113

[34] Xu C Zhang Y Liu D Shen R Razavi P Liu S P1.01-99 detecting HER2 alterations by Next Generation Sequencing (NGS) in patients with advanced NSCLC from the United States and China. J Thora Oncol. (2018) 13:S502 10.1016/j.jtho.2018.08.655

[35] McGranahan N Favero F de Bruin EC Birkbak NJ Szallasi Z Swanton C. Clonal status of actionable driver events and the timing of mutational processes in cancer evolution. Sci Transl Med. (2015) 7:283ra54. 10.1126/scitranslmed.aaa1408 25877892 PMC4636056

[36] Rachiglio AM Fenizia F Piccirillo MC Galetta D Crino L Vincenzi B. The presence of concomitant mutations affects the activity of EGFR tyrosine kinase inhibitors in EGFR-mutant non-small cell lung cancer (NSCLC) patients. Cancers. (2019) 11:341. 10.3390/cancers11030341 30857358 PMC6468673

[37] McGranahan N Swanton C. Biological and therapeutic impact of intratumor heterogeneity in cancer evolution. Cancer Cell. (2015) 27:15–26. 10.1016/j.ccell.2014.12.001 25584892

[38] Jamal-Hanjani M Hackshaw A Ngai Y Shaw J Dive C Quezada S. Tracking genomic cancer evolution for precision medicine: the lung TRACERx study. PLoS Biol. (2014) 12:e1001906. 10.1371/journal.pbio.1001906 25003521 PMC4086714

[39] van der Wekken AJ Saber A Hiltermann TJ Kok K van den Berg A Groen HJ. Resistance mechanisms after tyrosine kinase inhibitors afatinib and crizotinib in non-small cell lung cancer, a review of the literature. Crit Rev Oncol Hematol. (2016) 100:107–16. 10.1016/j.critrevonc.2016.01.024 26852079

[40] Takezawa K Pirazzoli V Arcila ME Nebhan CA Song X de Stanchina E. HER2 amplification: a potential mechanism of acquired resistance to EGFR inhibition in EGFR-mutant lung cancers that lack the second-site EGFRT790M mutation. Cancer Dis. (2012) 2:922–33. 10.1158/2159-8290.CD-12-0108 22956644 PMC3473100

[41] Rotow J Bivona TG. Understanding and targeting resistance mechanisms in NSCLC. Nat Rev Cancer. (2017) 17:637–58. 10.1038/nrc.2017.84 29068003

[42] Chuang JC Stehr H Liang Y Das M Huang J Diehn M. ERBB2-mutated metastatic non-small cell lung cancer: response and resistance to targeted therapies. J Thorac Oncol. (2017) 12:833–42. 10.1016/j.jtho.2017.01.023 28167203 PMC5402884

