# Supplementary material for: Co-Occurring Alterations of ERBB2 Exon 20 Insertion in Non-Small Cell Lung Cancer (NSCLC) and the Potential Indicator of Response to Afatinib
Source: Front Oncol. 2020 May 12;10:729. doi: 10.3389/fonc.2020.00729 (PMC7236802; doi:10.3389/fonc.2020.00729)
Supplement: Supplementary file 2 [file Data_Sheet_1.docx]

**Supplemental Online Methods**

DNA extraction

Circulating DNA was isolated from 3ml of plasma or effusion using the QIAamp Circulating Nucleic Acid Kit (Qiagen, Hilden, Germany) and peripheral blood lymphocytes (PBL) DNA were extracted using the DNeasy Blood & Tissue Kit (Qiagen, Hilden, Germany) for germline reference. Genomic DNA was extracted from FFPE samples using Maxwell® RSC DNA FFPE Kit (Promega, Madison, WI, USA). DNA concentration was measured by a Qubit fluorometer (Invitrogen, Carlsbad, VA USA) and the Qubit dsDNA HS (High Sensitivity) Assay Kit (Invitrogen, Carlsbad, CA, USA).
