# Supplementary material for: Co-Occurring Alterations of ERBB2 Exon 20 Insertion in Non-Small Cell Lung Cancer (NSCLC) and the Potential Indicator of Response to Afatinib
Source: Front Oncol. 2020 May 12;10:729. doi: 10.3389/fonc.2020.00729 (PMC7236802; doi:10.3389/fonc.2020.00729)
Supplement: Supplementary file 3 [file Data_Sheet_2.docx]

**Supplementary materials**

**Supplementary tables**

**Table S2:** **Insertion-subtype abundance among different sample types**

|  | A775ins | G776indel | P780ins | Total | Co-amp | *P* value |
| --- | --- | --- | --- | --- | --- | --- |
| Tissue | 41 | 11 | 9 | 61 | 2 | 0.411 |
| Plasma | 34 | 7 | 3 | 44 | 0 |  |
| Effusion | 4 | 1 | 2 | 7 | 0 |  |

*P* values are calculated using Fisher’s exact test among A775ins, G776indel, G780ins.

Abbreviation: ins: insertion; indel: deletion and insertion; Co-amp: Co-amplification

**Table S3: Exon distribution of TP53 mutation in three insertion-site subtypes**

| **Subtype** | **Ex4** | **Ex5** | **Ex6** | **Ex7** | **Ex8** | **Ex9** | **Ex10** | **Ex11** | **Intron** |
| --- | --- | --- | --- | --- | --- | --- | --- | --- | --- |
| **Data from our cohort** | | | | |  |  |  |  |  |
| **A775**  **(n=50)** | 3 (6.0%) | 14  (28.0%) | 9  (18.0%) | 7  (14.0%) | 10  (20.0%) | 0  (0.0%) | 4  (8.0%) | 0  (0.0%) | 3  (6.0%) |
| **G776**  **(n=13)** | 0  (0.0%) | 4  (30.7%) | 2  (15.4%) | 1  (7.7%) | 1  (7.7%) | 0  (0.0%) | 0  (0.0%) | 0  (0.0%) | 5  (38.5%) |
| **P780**  **(n=11)** | 1  (9.1%) | 2  (18.2%) | 1  (9.1%) | 2  (18.2%) | 2  (18.2%) | 2  (18.2%) | 0  (0.0%) | 1  (9.1%) | 0  (0.0%) |
| **Total** | **4** | **20** | **12** | **10** | **13** | **2** | **4** | **1** | **8** |
| **(n=74)** | （5.4%） | （27%） | （16.2%） | （13.5%） | （17.6%） | （2.7%） | （5.4%） | （1.4%） | （10.8%） |
| **Data from MSKCC cohort** | | | |  |  |  |  |  |  |
| **Total 2 3 7** | | | | **6** | **2** | **1** | **0** | **0** | **1** |
| **(n=22) （9.1%） (13.7%) (31.8%)** | | | | **(27.3%)** | **(9.1%)** | **(4.5%)** | **(0.0%)** | **(0.0%)** | **(4.5%)** |

**Table S4:** **Co-occurring genes classified by biological pathway involved in patients with ERBB2 exon 20 insertion between Our Cohort and MSKCC Cohort**

| **Signaling Pathway** | **Co-occurring genes** | **Our Cohort (%)** | **MSKCC Cohort (%)** | ***P* value** |
| --- | --- | --- | --- | --- |
| **P53 Pathway** | **P53** | **74(66.1%)** | **20 (66.7%)** | **0.951** |
| ***Cell cycle** | RB1 | 9 | 2 |  |
|  | CDKN2A | 6 | 7 |  |
|  | CDK4 | 4 | 2 |  |
|  | CCND1 | 2 | 0 |  |
|  | **Sum** | **20 (17.9%)** | **11 (36.7%)** | **0.044*** |
| **Receptor tyrosine kinase/growth factor signaling (RTK)** | MET | 4 | 0 |  |
|  | FGFR1 | 3 | 0 |  |
|  | ROS1 | 2 | 0 |  |
|  | RET | 2 | 0 |  |
|  | NTRK1 | 2 | 1 |  |
|  | ALK | 2 | 0 |  |
|  | PDGFRA | 1 | 0 |  |
|  | PTPN11 | 1 | 1 |  |
|  | DDR2 | 1 | 1 |  |
|  | FLT3 | 1 | 0 |  |
|  | FGFR2 | 1 | 0 |  |
|  | EGFR | 1 | 0 |  |
|  | **Sum** | **17 (15.2%)** | **2 (6.7%)** | **0.365** |
| **DNA Damage/Repair** | ATM | 5 | 0 |  |
|  | BRCA2 | 3 | 1 |  |
|  | BRCA1 | 3 | 0 |  |
|  | MLH1 | 1 | 0 |  |
|  | **Sum** | **10 (8.9%)** | **1 (3.3%)** | **0.458** |
| **PI3K/AKT1/MTOR** | PTEN | 3 | 1 |  |
|  | PIK3CA | 4 | 1 |  |
|  | TSC2 | 2 | 0 |  |
|  | TSC1 | 2 | 1 |  |
|  | AKT1 | 2 | 0 |  |
|  | MTOR | 1 | 0 |  |
|  | **Sum** | **13 (11.6%)** | **3 (10%)** | **1** |
| **RAS-RAF-MAPK** | NF1 | 6 | 1 |  |
|  | BRAF | 3 | 0 |  |
|  | **Sum** | **9 (8.0%)** | **1 (3.3%)** | **0.689** |
| **Hedgehog signaling pathway** | SMO | 2 | 0 |  |
|  | PTCH1 | 3 | 1 |  |
|  | **Sum** | **4 (3.6%)** | **1 (3.3%)** | **1** |
| **β-catenin/Wnt signaling** | APC | 3 | 1 |  |
|  | CTNNB1 | 2 | 1 |  |
|  | **Sum** | **5 (4.5%)** | **2 (6.7%)** | **0.639** |
| **Chromatin remodeling/DNA Methylation** | **MSH6** | **3(2.7%)** |  | **nc** |
| **MAP kinase signaling** | SRC | 2 |  |  |
|  | MAP2K1 | 1 |  |  |
|  | **Sum** | **3(2.7%)** |  | **nc** |
| **JAK/STAT signaling** | **JAK2** | **2 (1.8%)** |  | **nc** |

*P* values are calculated using Fisher’s exact test. * represents the pathway significantly different between two cohorts.

The total number not equal to the respective categorical counts is due to some of the patients having multiple gene alterations in a single pathway.

Abbreviation: nc: not calculate.

**Table S5:** **Comparison of the proportion of ERBB2ex20ins subtypes and the molecular co-occurring spectrum between Our Cohort and MSKCC Cohort**

| **Characteristic** | **Our Cohort** | **MSK Cohort(n=30)** | ***P* value** |
| --- | --- | --- | --- |
| **Total cases, N** | 112 | 30 |  |
| **ERBB2ex20ins subtypes** | | | |
| A775 insertion | 79(70.5%) | 21(70.0%) | 0.450 |
| G776 indel | 19(17.0%) | 3(10.0%) |  |
| P780 insertion | 14(12.5%) | 6(20.0%) |  |
| **Co-occurring alterations in 59 Panel** | | | |
| *ERBB2 amplification | 2(1.8%) | 4(13.3%) | 0.018* |
| TP53 alteration | 74(66.1%) | 20(66.7%) | 0.951 |
| RB1 alteration | 9(8.0%) | 2(6.7%) | 1 |
| NF1 alteration | 6(5.4%) | 1(3.3%) | 1 |
| *CDKN2A alteration | 6(5.4%) | 7(23.3%) | 0.007* |
| CDK4 alteration | 4(3.6%) | 2(6.7%) | 0.607 |
| **Co-occurring alterations in 1021 Panel** | | | |
| **Total cases, N** | 55 | 30 | *P* Value |
| MLL3 alteration | 5(9.1%) | 2(6.7%) | 1 |
| EPHA5 alteration | 5(9.1%) | 1(3.3%) | 0.417 |
| TERT alteration | 4(7.3%) | 5(16.7%) | 0.268 |
| CDK12 alteration | 4(7.3%) | 3(10%) | 0.693 |
| *FOXA1 alteration | 3(5.5%) | 7(23.3%) | 0.029* |
| FAT1 alteration | 3(5.5%) | 1(3.3%) | 1 |
| FGFR4 alteration | 3(5.5%) | 1(3.3%) | 1 |
| TET2 alteration | 3(5.5%) | 1(3.3%) | 1 |
| KDR alteration | 3(5.5%) | 0(0.0%) | 0.549 |
| NOTCH1 alteration | 3(5.5%) | 0(0.0%) | 0.549 |
| *CDKN2B alteration | 0(0.0%) | 5(16.7%) | 0.004* |
| *RBM10 alteration | 0(0.0%) | 3(10.0%) | 0.041* |
| PIK3CG alteration | 2(3.6%) | 2(6.7%) | 0.611 |
| ATR alteration | 1(1.8%) | 2(6.7%) | 0.283 |
| EPHA3 alteration | 1(1.8%) | 2(6.7%) | 0.283 |
| MED12 alteration | 1(1.8%) | 2(6.7%) | 0.283 |
| MYC alteration | 1(1.8%) | 2(6.7%) | 0.278 |
| CHEK2 alteration | 1(1.8%) | 2(6.7%) | 0.278 |
| ETV1 alteration | 0(0.0%) | 2(6.7%) | 0.122 |
| GNAS alteration | 0(0.0%) | 2(6.7%) | 0.122 |
| RAD50 alteration | 0(0.0%) | 2(6.7%) | 0.122 |
| SDHA alteration | 0(0.0%) | 2(6.7%) | 0.122 |
| RARA alteration | 0(0.0%) | 2(6.7%) | 0.122 |

*P* values are calculated using Fisher’s exact test.

* represent the frequency was significantly different between two cohorts.

**Table S6:** **Evaluation of the impact of ERBB2 insertion-site subtypes and co-occurring alterations on afatinib treatment outcome**

| Variable | Multivariable | | |
| --- | --- | --- | --- |
|  | HR (95%CI) | *P* Value | |
| **Insertion subtype** |  |  |  |
| A775ins | 1 | | 0.187 |
| G776indel | 0.105 (0.008-1.365) | | 0.085 |
| P780ins | 0.322 (0.026-4.044) | | 0.380 |
| ***Clonality Status** |  |  |  |
| Subclonal | 1 | | 0.01* |
| Clonal | 0.025 (0.002-0.41) | |  |
| **Co-TP53 mutation** |  |  |  |
| No | 1 | | 0.655 |
| Yes | 1.68 (0.17-16.47) | |  |
| **Co-TP53mis mutation** |  |  |  |
| No | 1 | | 0.687 |
| Yes | 0.646 (0.077-5.423) | |  |
| **No. line of treatment** |  |  |  |
| 1 | 1 | | 0.435 |
| ≥2 | 0.531 (0.108-2.599) | |  |

*represent the factor with significant prognosis value.

Abbreviation: Co-TP53 mutation: Co-occurring TP53 mutation; Co-TP53mis mutation: Co-occurring TP53 missense mutation.

**Table S7:** **Clinical characteristics and test details for two patients with dynamic detection**

|  | Patient#1 | Patient#2 |
| --- | --- | --- |
| **Basic Characteristic** | | |
| Age at diagnosis | 56 | 62 |
| Gender | Female | Female |
| Smoking status | Never | Never |
| Histology | Adenocarcinoma | Adenocarcinoma |
| Stage | IV | IV |
| ERBB2 alteration | p. P780_Y781insGSP | p.A775_G776insVVMA |
| Best response to afatinib | SD | SD |
| PFS of afatinib | 2.5 months | 4.9 months |
| **First NGS details (59 panel)** |  |  |
| Time | Before afatinib treatment | Before afatinib treatment |
| Sample type | Biopsy tumor tissue | Plasma sample |
| Other alterations | TP53 p.Q317* | TP53 p. Y234N  CDKN2A p. R58*  MET splice-5 |
| **Second NGS details (59 panel)** | | |
| Time | Progression on afatinib | afatinib treated for 1/2 months |
| Sample type | Biopsy tumor tissue | Plasma sample |
| Other alterations | TP53 p. Q317*  ERBB2 amp; CN=3.1 | TP53 p. Y234N  CDKN2A p. R58*  MET splice-5  ERBB2amp; CN=2.74 |

Abbreviation: amp: amplification; CN: copy number

**Supplementary figures**

**
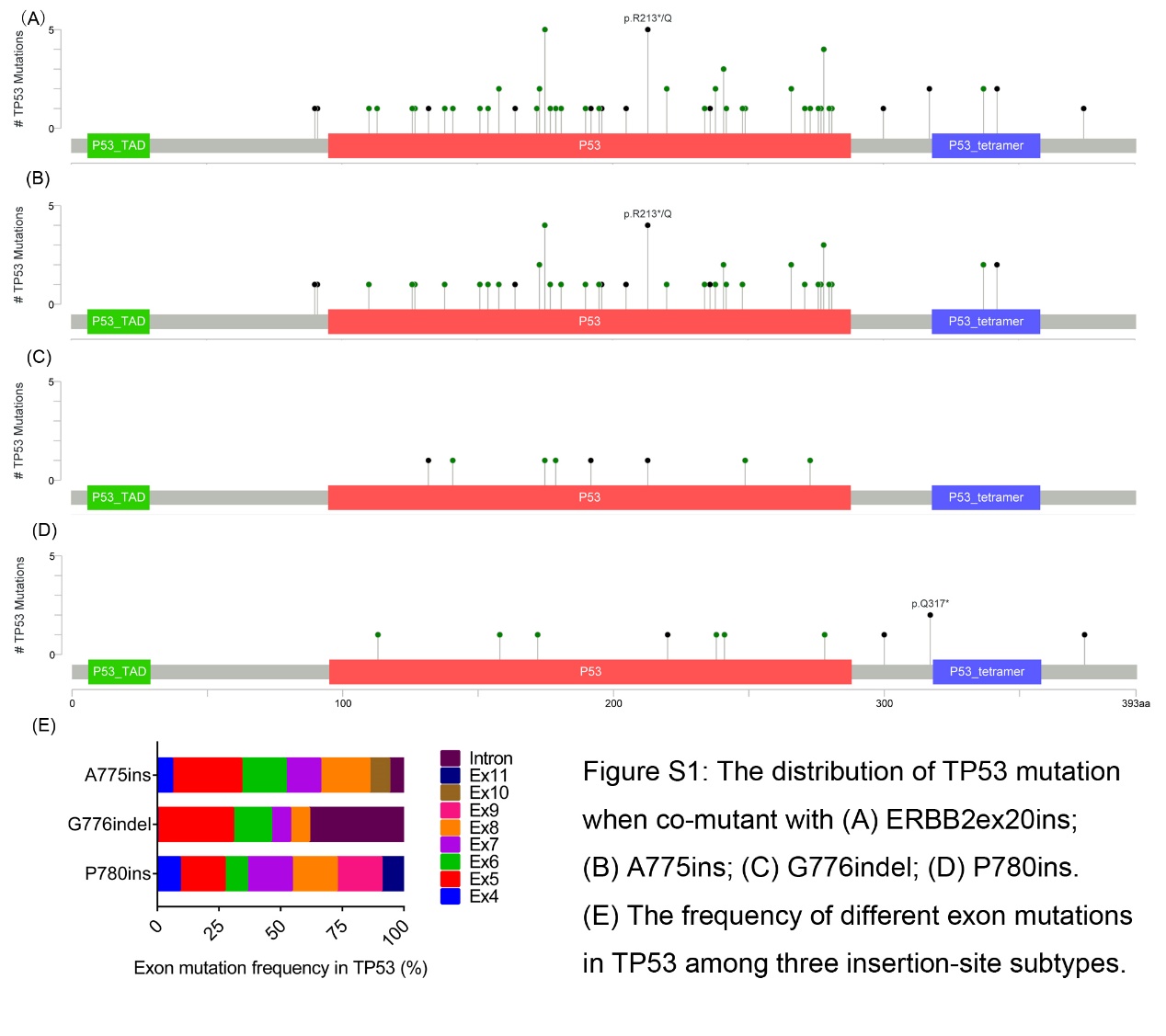
**


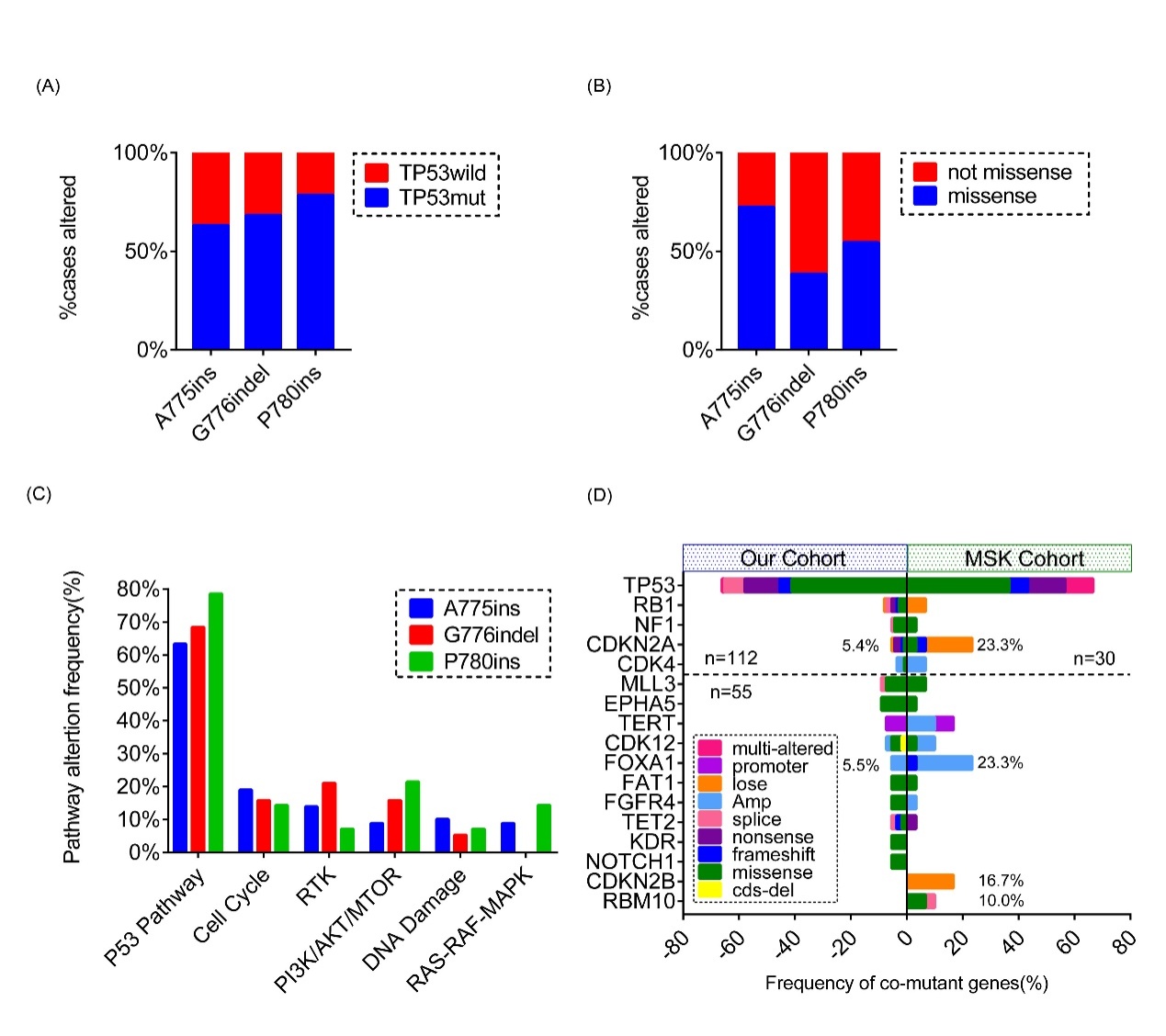


**Figure S2:** Frequency of concurrent (A) TP53 mutation (Fisher’s exact test, *P*>0.05) and (B) TP53 missense mutation (Fisher’s exact test, *P*=0.06) among three insertion-site subtypes.

(C) Comparison of enriched pathway for co-occurring genes among three insertion-site subtypes; RTK, receptor tyrosine kinase/growth factor signaling

(D) The co-occurring genomic spectrum between our cohort and MSKCC cohort (only the genes in our cohort with a frequency greater than 5 cases were shown). The genes labeled with the accurate frequency were significantly different between the two cohorts.


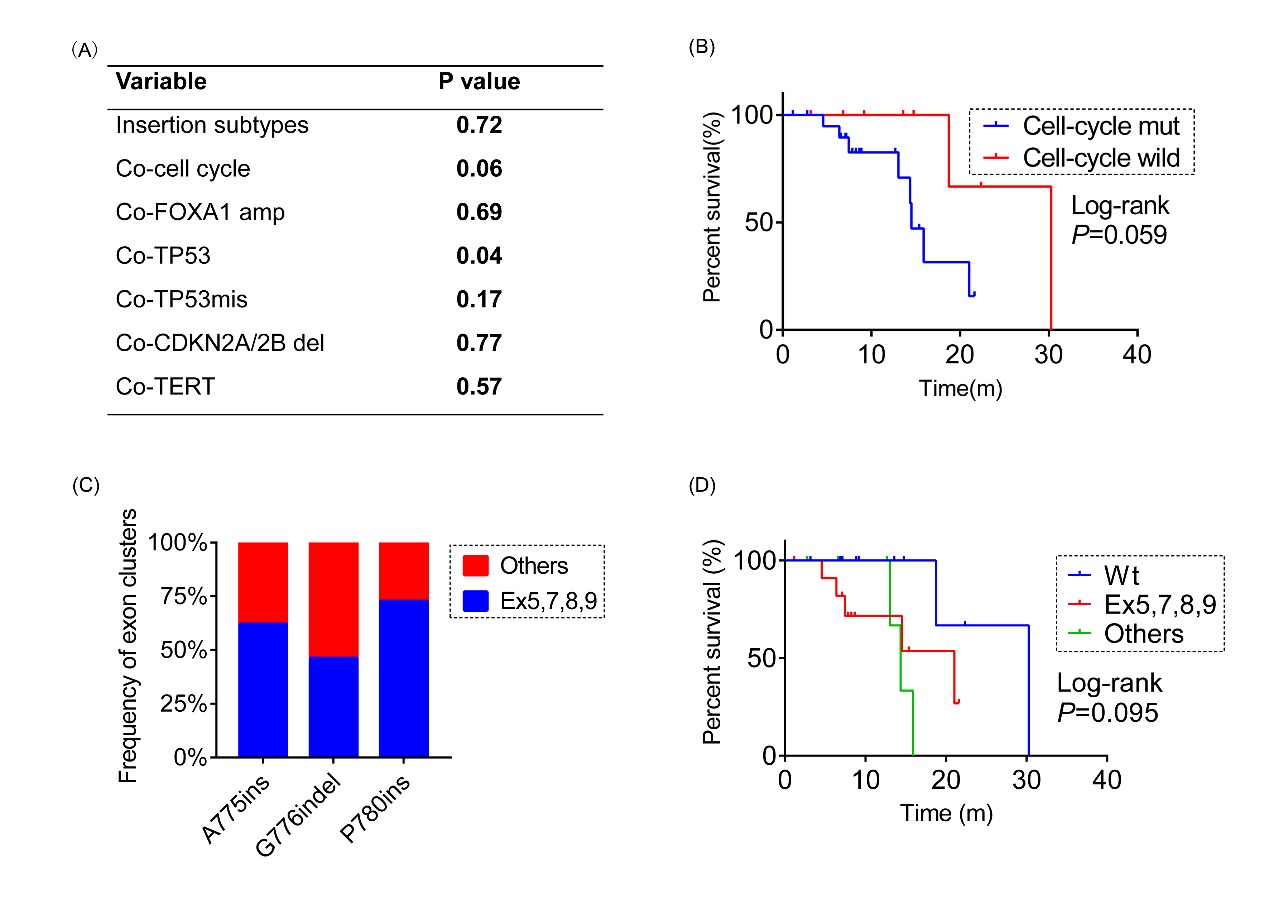


**Figure S3:** (A) Evaluation of the impact of ERBB2 insertion-site subtypes and co-occurring alterations on OS. *p* values are calculated using the log-rank test.

Abbreviation: Co-FOXA1 amp: Co-occurring FOXA1 amplification; Co-TP53mis: Co-occurring TP53 missense mutation; Co-CDKN2A/2B del: Co-occurring CDKN2A/2B deletion; Co-cell Cycle: Co-mutant genes enriched in the cell cycle pathway.

(B) Kaplan–Meier curve visualizing the effect of cell-cycle genes’ alteration on OS.

(C) Frequency of exon clusters (exons 5,7,8,9) of TP53 mutation in three insertion-site subtypes.

(D) Kaplan–Meier curve visualizing the effect of different exon clusters of TP53 alteration on the overall survival (Abbreviations: wt: TP53 wild type; Ex5,7,8,9: TP53 mutations located in the exon 5,7,8,9; Others: TP53 mutated in other exons or intron)
